# Acute Ischemic Stroke and Transient Ischemic Attack in ST-Segment Elevation Myocardial Infarction Patients Who Underwent Primary Percutaneous Coronary Intervention

**DOI:** 10.3390/jcm12030840

**Published:** 2023-01-20

**Authors:** Tsukasa Murakami, Kenichi Sakakura, Hiroyuki Jinnouchi, Yousuke Taniguchi, Takunori Tsukui, Yusuke Watanabe, Kei Yamamoto, Masaru Seguchi, Hiroshi Wada, Hideo Fujita

**Affiliations:** Division of Cardiovascular Medicine, Saitama Medical Center, Jichi Medical University, 1-847 Amanuma, Omiya, Saitama City 330-8503, Japan

**Keywords:** primary percutaneous coronary intervention, acute ischemic stroke, coronary angiography, transient ischemic attack

## Abstract

Background: Acute ischemic stroke (AIS) is a rare but critical complication following ST-elevation myocardial infarction (STEMI). The risk of AIS or transient ischemic attack (TIA) may be amplified by invasive procedures, including primary percutaneous coronary intervention (PCI). This study aimed to investigate the factors associated with in-hospital AIS/TIA in patients with STEMI who required primary PCI. Methods: We included 941 STEMI patients who underwent primary PCI and divided them into an AIS/TIA group (*n* = 39) and a non-AIS/TIA group (*n* = 902), according to new-onset AIS/TIA. The primary interest was to find the factors associated with AIS/TIA by multivariate logistic regression analysis. We also compared clinical outcomes between the AIS/TIA and non-AIS/TIA groups. Results: The incidence of in-hospital deaths was significantly higher in the AIS/TIA group (46.2%) than in the non-AIS/TIA group (6.3%) (*p* < 0.001). Multivariate analysis revealed that cardiogenic shock (OR 3.228, 95% CI 1.492–6.986, *p* = 0.003), new-onset atrial fibrillation (AF) (OR 2.280, 95% CI 1.033–5.031, *p* = 0.041), trans-femoral approach (OR 2.336, 95% CI 1.093–4.992, *p* = 0.029), use of ≥4 catheters (OR 3.715, 95% CI 1.831–7.537, *p* < 0.001), and bleeding academic research consortium (BARC) type 3 or 5 bleeding (OR 2.932, 95% CI 1.256–6.846, *p* = 0.013) were significantly associated with AIS/TIA. Conclusion: In STEMI patients with primary PCI, new-onset AIS/TIA was significantly associated with cardiogenic shock, new-onset AF, trans-femoral approach, the use of ≥4 catheters, and BARC type 3 or 5 bleeding. We should recognize these modifiable and unmodifiable risk factors for AIS/TIA in the treatment of STEMI.

## 1. Introduction

Acute ischemic stroke (AIS) is a rare but critical complication following acute myocardial infarction (AMI) [1,2,3]. Several clinical variables, including age, gender, ST-segment elevation myocardial infarction (STEMI), atrial fibrillation (AF), and specific interventional procedures have been reported as risk factors for acute stroke in AMI patients [1,4,5,6,7]. Among those factors, specific interventional procedures may be more important than unmodifiable factors such as age or gender, because physicians may have an opportunity to modify their interventional procedures to prevent AIS. Although earlier studies reported that transfemoral intervention, thrombus aspiration, and mechanical support were associated with stroke in patients with percutaneous coronary intervention (PCI), interventional procedures associated with AIS in patients with STEMI have not been fully discussed [7,8,9]. The incidence of AIS or transient ischemic attack (TIA) is greater in STEMI cases than in non-ST segment elevation myocardial infarction (NSTEMI) cases [4,5]. Furthermore, emergent coronary angiography (CAG) and primary PCI are definitely necessary for patients with STEMI [10,11]. This study aimed to investigate the factors associated with new-onset AIS/TIA in patients with STEMI who required primary PCI.

## 2. Materials and Methods

The present study is a retrospective, single-center study. We reviewed consecutive AMI patients who were admitted to the Saitama Medical Center, Jichi Medical University, from 1 January 2015 to 31 March 2022. The inclusion criterion was patients with STEMI. The exclusion criteria were (1) patients who did not undergo primary PCI to the culprit lesion within 24 h since arrival or in-hospital onset of STEMI, (2) patients who developed STEMI after the diagnosis of AIS or acute hemorrhagic stroke, (3) patients who developed STEMI because of acute type A aortic dissection, and (4) patients who developed STEMI because of coronary artery dissection due to CAG [12].

The final study population was divided into an AIS/TIA group and a non-AIS/TIA group, according to new-onset AIS/TIA during the index admission. The new-onset AIS/TIA was confirmed by both clinical assessment and brain imaging studies [computed tomography (CT) and/or magnetic resonance imaging (MRI)]. AIS was defined as sudden-onset neurologic deficit with new-onset ischemic lesions detected by brain imaging, while TIA was defined as transient neurologic deficit without new-onset ischemic lesions [13]. The day of the new-onset AIS/TIA was obtained from the hospital records. When the onset-day was not clinically determined, the day of the brain imaging was substituted as the onset-day. The primary interest was to find factors associated with new-onset AIS/TIA during the index admission. The secondary interest was to compare the clinical outcomes between the AIS/TIA and non-AIS/TIA groups. We acquired clinical information from hospital records. All procedures were carried out in accordance with the Declaration of Helsinki.

Since our institution is a teaching university hospital, most PCIs except complex ones are performed by senior residents with the supervision of staff interventional cardiologists [14]. We routinely use the 5 Fr Judkins right (JR) catheter and Judkins left (JL) catheter for CAG. In primary PCI, we routinely use Amplatz left (AL) or JR guiding catheters for the right coronary artery (RCA) and contralateral left support (CLS) or JL guiding catheters for the left coronary artery (LCA). As a result, the most typical number of catheters used in primary PCI was three (two diagnostic catheters and one guiding catheter). The choice of devices is at the discretion of the staff interventional cardiologist.

In the present study, AMI was defined according to the universal definition [15]. Diagnostic ST-segment elevation was defined as new ST elevation at the J point in at least two contiguous leads ≥2 mm (0.2 mV) in leads V2–V3 or ≥1 mm (0.1 mV) in all the rest of the leads, whereas all others were defined as not having ST-segment elevation [16,17]. The definition of hypertension, dyslipidemia, and diabetes mellitus were described in previous literature [16]. New-onset AF was defined as AF without a clinical history of AF prior to admission. Cardiogenic shock was defined as systolic blood pressure of <90 mmHg or the need for vasopressors to maintain blood pressure [18]. Hemorrhagic complications were defined by the bleeding academic research consortium (BARC) definition [19]. According to the BARC bleeding criteria, type 3 or 5 bleeding was classified as a major hemorrhagic complication [20]. Quantitative coronary angiography (QCA) parameters were measured using a cardiovascular angiography analysis system (QAngio XA 7.3, MEDIS Imaging Systems, Leiden, The Netherlands) [16,21,22]. All study lesions were classified into type A, B1, B2, or C, according to the ACC/AHA lesion-classification system [23]. Anomalous origin of coronaries was confirmed based on angiographic findings in CAG, which included RCA from the ascending aorta, RCA from the left sinus of Valsalva, LCA from the right sinus of Valsalva, separate origin of the left anterior descending artery (LAD) and the left circumflex artery (LCX), and LCA from the ascending aorta [24].

## 3. Statistical Analysis

Data are presented as a percentage for categorical variables, a mean ± standard deviation for normally distributed continuous variables, or a median and inter-quartile range for non-normally distributed continuous variables. Categorical variables were compared by Fischer’s exact test. The Wilk–Shapiro test was performed to determine whether the continuous variables were normally distributed. Normally distributed continuous variables were compared by the unpaired Student’s *t*-test. Otherwise, continuous variables were compared by the Mann–Whitney U test. First, we compared the clinical and procedural characteristics between the AIS/TIA and non-AIS/TIA groups to identify the profiles of patients at increased risk of developing in-hospital AIS/TIA. Then, to investigate which variables were independently associated with new-onset AIS/TIA, multivariate logistic regression analysis with likelihood ratio statistical criteria using a backward elimination method was performed. We selected independent variables that showed a significant difference (*p* < 0.05) between the AIS/TIA and non-AIS/TIA groups. When there were ≥2 similar variables, only one variable was entered into the model, to avoid multi-collinearity. Regarding cardiogenic shock and out-of-hospital cardiac arrest (OHCA), we developed two models of multivariate analysis to investigate the independent factors for AIS/TIA. Model 1 included cardiogenic shock as the independent variable, whereas Model 2 included OHCA as the independent variable. A *p* value < 0.05 was considered statistically significant. We analyzed all data by SPSS ver. 28 for Windows (SPSS, Inc., Chicago, IL, USA).

## 4. Results

**Figure 1:**
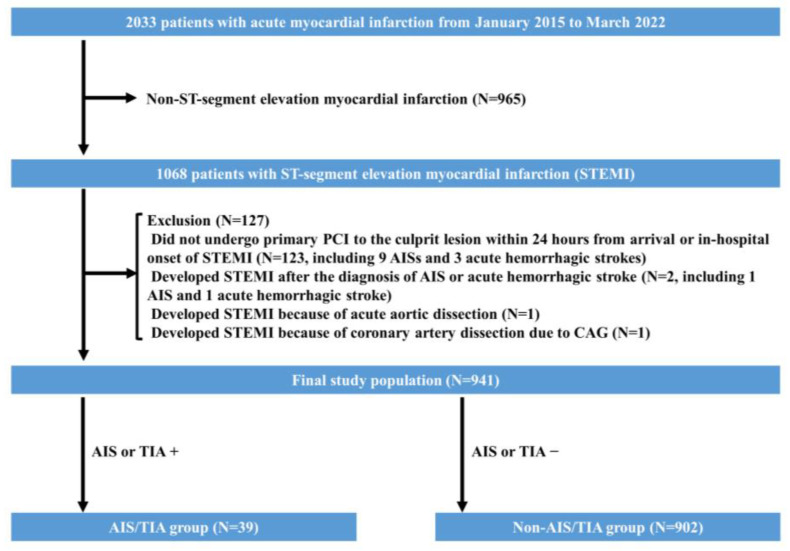
The study flow chart. Abbreviations: AIS = acute ischemic stroke, CAG = coronary angiography, PCI = percutaneous coronary intervention, STEMI = ST-segment elevation myocardial infarction, TIA = transient ischemic attack.

From January 2015 to March 2022, there were 1068 STEMI cases. We excluded 127 cases according to the exclusion criteria, in which there were 10 AISs and four acute hemorrhagic strokes. Accordingly, we included 941 cases as the final study population. Thirty-nine patients (4.1%) developed new-onset AIS/TIA during the index admission. We divided the final study population into an AIS/TIA group (*n* = 39) and a non-AIS/TIA group (*n* = 902). Of the 39 cases in the AIS/TIA group, 34 cases were AIS, two cases were AIS with hemorrhagic transformation, and three cases were TIA. No patients developed acute hemorrhagic stroke during the study period. The study flowchart is shown in Figure 1.

The comparisons of clinical, lesion, and procedural characteristics between the AIS/TIA and non-AIS/TIA groups are shown in Table 1 and Table 2. The prevalence of cardiogenic shock, OHCA, and the incidence of new-onset AF were higher in the AIS/TIA group than in the non-AIS/TIA group. The use of ≥4 catheters was more frequently observed in the AIS/TIA group than in the non-AIS/TIA group. Triple vessels disease, final TIMI flow grade ≤2, trans-femoral approach, size of guiding catheter ≥7 Fr, V-A ECMO during primary PCI, Impella during primary PCI, and BARC type 3 or 5 bleeding were more frequently observed in the AIS/TIA group than in the non-AIS/TIA group.

**Figure 2:**
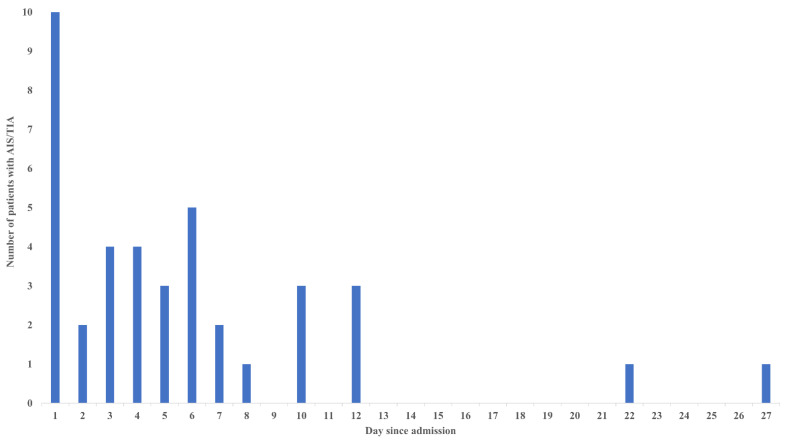
The distribution of the onset-day of AIS/TIA. The peak period of the occurrence of AIS/TIA was within 24 h of admission. Abbreviations: AIS = acute ischemic stroke, TIA = transient ischemic attack.

The distribution of the onset-day of AIS/TIA is shown in Figure 2. Of 39 AIS/TIA cases, 30 AIS/TIA (76.9%) occurred within 7 days of admission. Notably, the peak period of new-onset AIS/TIA was within 24 h of admission. The characteristics of 39 AIS/TIA cases are described in Supplementary Table S1. Of the 39 AIS/TIA cases, 19 (48.7%) patients developed multiple cerebral infarctions. The comparison of clinical outcomes between the AIS/TIA and non-AIS/TIA groups is shown in Table 3. The incidence of in-hospital death was greater in the AIS/TIA group (46.2%) than in the non-AIS/TIA group (6.3%) (*p* < 0.001) The association between AIS/TIA and high mortality was significant both in patients with and without cardiogenic shock. In addition, we divided the AIS/TIA cases into the two groups according to the onset time: an early-onset group (AIS/TIA within 72 h) and a late-onset group (AIS/TIA beyond 72 h). We performed additional analysis to compare the clinical characteristics and outcomes between the early-onset AIS/TIA group and the late-onset AIS/TIA group to further understand the mechanism of AIS/TIA (Supplementary Table S2). The number of used catheters in primary PCI was significantly greater in the early-onset AIS/TIA group [4 (3–4)] than in the late-onset AIS/TIA group [3 (3–4)] (*p* = 0.048).

The multivariate stepwise logistic regression analysis is shown in Table 4. An initial Model 1 included cardiogenic shock, new-onset AF, lesion eccentricity, trans-femoral approach, use of ≥4 catheters, BARC type 3 or 5 bleeding, estimated GFR, triple vessels dis-ease, final TIMI flow grade ≤2, size of guiding catheters ≥7 Fr, and multivessel PCI at the time of primary PCI as independent variables. An initial Model 2 included OHCA instead of cardiogenic shock and the remaining independent variables were same as those in Model 1. In Model 1, cardiogenic shock (OR 3.228, 95% CI 1.492–6.986, *p* = 0.003), new-onset AF (OR 2.280, 95% CI 1.033–5.031, *p* = 0.041), trans-femoral approach (OR 2.336, 95% CI 1.093–4.992, *p* = 0.029), use of ≥4 catheters (OR 3.715, 95% CI 1.831–7.537, *p* < 0.001), and BARC type 3 or 5 bleeding (OR 2.932, 95% CI 1.256–6.846, *p* = 0.013) were significantly associated with new-onset AIS/TIA, after controlling for confounding factors. In Model 2, OHCA was significantly associated with AIS/TIA and the remaining independent factors were same as those in Model 1.

## 5. Discussion

Of the 941 patients with STEMI who underwent primary PCI, 39 (4.1%) patients developed new-onset AIS/TIA during the index admission. Of the 39 new-onset AIS/TIA patients, 76.9% of the cases occurred within 7 days; notably, the first 24 h was the peak period. New-onset AIS/TIA was associated with poor clinical outcomes. The multivariate logistic analysis revealed that cardiogenic shock, OHCA, new-onset AF, trans-femoral approach, use of ≥4 catheters, and BARC type 3 or 5 bleeding were significantly associated with new-onset AIS/TIA.

The incidence of new-onset AIS/TIA tended to be higher in our study (4.1%) than in earlier studies, partly because the median age of patients with STEMI was older in our study (71 years) than in earlier studies [3,7,25]. In addition, the prevalence of cardiogenic shock was higher in our study (15.9%) than in earlier studies (2–10%) [3,7,25]. Accoding to the Japanese multi-center registry, the prevalence of cardiogenic shock following STEMI was 16%, increasing with the times [26]. Higher age and higher prevalence of cardiogenic shock can be causes of the higher incidence of AIS/TIA in our study. Because we did not perform brain imaging for patients who were asymptomatic, we might have missed asymptomatic acute AIS/TIA, as was the case in earlier studies [7,25]. Interestingly, Murai et al. conducted routine head MRIs for 75 patients with ACS and revealed that 34.7% of patients with PCI developed asymptomatic AIS [27], suggesting that asymptomatic AIS was missed in both our cohort and earlier cohorts [7,25]. Primary PCI has the potential to induce ischemic stroke, whether symptomatic or not.

Along with trans-femoral approach and use of ≥4 catheters, triple vessel disease, multivessel PCI, size of guiding catheters ≥7 Fr, and V-A ECMO were more frequently observed in the AIS/TIA group than in the non-AIS/TIA group. These results suggest that procedural complexity might be associated with the occurrence of AIS/TIA. We also revealed that some angiographic features, including lesion eccentricity and final TIMI flow grade ≤2, were more frequently observed in the AIS/TIA group than in the non-AIS/TIA group. In addition, the prevalence of cardiogenic shock, OHCA, new-onset AF, and BARC type 3 or 5 bleeding was higher in the AIS/TIA group than in the non-AIS/TIA group. After subjecting these confounding factors to multivariate analysis, we determined five independent risk factors, including cardiogenic shock, new-onset AF, trans-femoral approach, use of ≥4 catheters, and BARC type 3 or 5 bleeding.

Earlier studies reported that triple vessel disease and intracoronary thrombus were the risk factors for the development of stroke [28]. However, in the present study, the association between angiographic features and AIS/TIA was not statistically significant from our multivariate analysis, partly because of the small number of AIS/TIA events. Of note, the use of many catheters (≥4 catheters) and trans-femoral approach in primary PCI would be associated with procedural complexity. If procedural complexity is associated with neurologic events, it would be reasonable to select simple procedures in primary PCI to prevent neurologic events.

Earlier studies reported that most periprocedural strokes occurred within 48 h of PCI [29,30]. Our results also showed that the first 24 h was the most vulnerable period for new-onset AIS/TIA, which suggests that catheter procedures would be an important cause of new-onset AIS/TIA in patients with STEMI. In addition, we revealed that the number of catheters used in primary PCI was greater in the early-onset AIS/TIA group than in the late-onset AIS/TIA group, suggesting that procedures of primary PCI might be one of the causes of early-onset AIS/TIA [29]. Thus, the number of catheters used may be a strong risk factor for AIS/TIA in patients with STEMI who undergo primary PCI. Hoffman et al. suggested that the use of many catheters was associated with PCI-related ischemic stroke in both emergent and elective PCI [31]. Although their study population was substantially different from our study population, our results were consistent with their findings.

Although the mechanisms of stroke were not elucidated in our study, atherosclerotic lesions of thoracic aorta might be a potential etiology of ischemic stroke [32,33]. A retrograde aortic flow, as well as an antegrade aortic flow, might cause ischemic stroke by disseminating dislodged aortic plaque. A study using time-resolved contrast-enhanced 3-dimensional MR angiography revealed that aortic plaque located at the upper thoracic aorta below the aortic arch had opportunities to reach all supra-aortic arteries retrogradely [33]. In addition, the insertion of catheters, especially large-lumen catheters, can scrape the aortic plaque by the gap between the guide catheter lumen and leading 0.035-inch guidewire [34,35]. Moreover, aortic plaque might become more vulnerable in patients with STEMI, compared with patients with stable angina or non-ST-segment elevation myocardial infarction [36]. The more catheters were used in primary PCI, the more frequently the aortic plaque was scraped by the gap between the guide catheter lumen and guidewire, which might result in disseminating embolic debris both anterogradely and retrogradely.

We showed the significant association between trans-femoral approach and AIS/TIA in the present study. However, the association between trans-femoral approach and AIS/TIA was not proven in the randomized trial, partly because of the small number of stroke cases [37]. Meanwhile, Shoji et al. showed that trans-femoral approach was significantly associated with periprocedural stroke after controlling for confounding factors [8]. Although their study included both acute coronary syndrome and chronic coronary syndrome, our results were consistent with their study. Compared to the ascending aorta, greater plaque is observed in the aortic arch and the descending aorta [38,39]. Kojima et al. showed that in patients with coronary artery disease, vulnerable plaque was frequently observed from the aortic arch to the common iliac artery by aortic angioscopy [40]. Compared to trans-radial approach, catheters from femoral artery can damage the greater aortic plaque from the iliac artery to the ascending aorta by the gap between the guide catheter lumen and guidewire in inserting or exchanging catheters, which may result in subsequent embolization.We revealed that 48.7% of AIS/TIA cases showed multiple cerebral infarctions by cerebral imaging studies, which is probably explained by embolization.

Some patient factors, such as cardiogenic shock, new-onset AF, and BARC type 3 or 5 bleeding. can be classified as unmodifiable risk factors for new-onset AIS/TIA. In earlier studies, cardiogenic shock and new-onset AF concomitant with acute myocardial infarction were associated with stroke [3,41]. Our findings were consistent with these earlier studies. In cases with cardiogenic shock, cerebral hypoperfusion would result in the occurrence of AIS/TIA. When new-onset AF was observed during hospitalization, patients with STEMI who already had dual-antiplatelet therapy might not receive additional anticoagulation therapy. The association between BARC type 3 or 5 bleeding and AIS/TIA may be partly explained by the discontinuation of antithrombotic therapy. In Table 2, we show that the prevalence of discontinuation of antithrombotic therapy was higher in the AIS/TIA group than in the non-AIS/TIA group. Furthermore, patients with high bleeding risk tend to have a greater risk of thrombotic events [42]. In the present study, patients with major bleeding might have both high bleeding risk and high ischemic risk.

Clinical implications in the present study should be noted. Since patients with cardiogenic shock are obviously sick, those patients are usually followed up carefully in intensive care units or coronary care units. New-onset AF should be recognized as a high-risk feature for AIS/TIA, and the introduction of anticoagulant therapy followed by the cessation of aspirin, with appropriate timing, should be considered once new-onset AF is detected. However, the use of many catheters (≥4 catheters), trans-femoral approach, and major bleeding have not been recognized as high-risk features for new-onset AIS/TIA in patients with STEMI. It may be useful to share this information with physicians and other medical staff, because early detection of AIS is closely associated with good neurological outcomes [43].

Furthermore, to reduce the risk of developing AIS/TIA, we should pay attention to the total number of catheters in primary PCI. Lee et al. demonstrated the usefulness of a single-catheter PCI strategy by using a universal catheter (Ikari left) in primary PCI for STEMI [44]. A single-catheter PCI strategy, if possible, may be reasonable to reduce new-onset AIS in primary PCI. In addition, if we perform diagnostic coronary angiography to the non-culprit artery first, followed by diagnostic coronary angiography to the culprit artery using a guiding catheter, we can reduce the average number of catheters used from three to two [45]. Regarding trans-femoral approach, which was another modifiable factor, we should consider using trans-radial approach first, even if patients are in cardiogenic shock. In comparison with trans-femoral approach, trans-radial approach in cardiogenic shock is related to lower incidences of in-hospital death and access-site bleeding [46]. Ultrasound guidance would be useful when radial artery puncture is difficult [47]. We showed that cardiogenic shock and BARC type 3 or 5 bleeding were associated with AIS/TIA. Avoiding trans-femoral approach may result in the reduced incidence of AIS/TIA thorough direct effect (less damage to aortic plaque and subsequent dissemination) and indirect effect (reduction of bleeding complication). When trans-femoral approach is selected, it would be better to use small lumen catheters, because the gap between the large catheter’s lumen and the guidewire may scrape the vulnerable plaque from the aortic arch and the descending aorta.

The present study has the following limitations. First, we might have missed potential AIS/TIA cases when brain imaging was not available. We might also have missed asymptomatic ischemic stroke because we defined the AIS/TIA as symptomatic cases with a diagnostic finding of head imaging. Although we tried to consult with neurologists for the diagnosis of AIS/TIA, not all of the AIS/TIA cases underwent clinical evaluation by neurologists. Second, we could not determine the AIS/TIA subtypes, such as thrombosis, embolism, and hypoperfusion. Third, since this study was a retrospective single-center study, there was a selection bias. Fourth, since the number of AIS/TIA was small, the multivariate logistic regression analysis could not include sufficient numbers of variables in the final model. Fifth, compared with IABP and V-A ECMO, the number of Impella was small in our institution, and this has been reported to be associated with several complications, including stroke [48]. Sixth, we counted the cases with transition from radial access to femoral access as trans-femoral approach, which might have resulted in a greater incidence of AIS/TIA in trans-femoral approach than trans-radial approach. Finally, our definition of cardiogenic shock did not include the biomarker evidence of hypoperfusion, such as high serum lactate, which may be a cause of the relatively higher prevalence of cardiogenic shock in our cohort. However, in the setting of STEMI with low blood pressure, immediate invasive procedures, including insertion of mechanica support, are sometimes inevitable to rescue otherwise fatal cases.

## 6. Conclusions

In patients with STEMI who underwent primary PCI, new-onset AIS/TIA was significantly associated with cardiogenic shock, new-onset AF, trans-femoral approach, the use of ≥4 catheters in primary PCI, and BARC type 3 or 5 bleeding. We should recognize these modifiable and unmodifiable risk factors for new-onset AIS/TIA for better clinical outcomes in patients with STEMI.

## Figures and Tables

**Table 1:** Comparison of clinical characteristics between the AIS/TIA group and the non-AIS/TIA group.

	All (*n* = 941)	AIS/TIA Group (*n* = 39)	Non-AIS/TIA Group (*n* = 902)	*p*-Value
Age, year	71 (61–79)	74 (68–77)	71 (61–79)	0.494
Male, *n* (%)	736 (78.2)	33 (84.6)	703 (77.9)	0.428
Body mass index >25 (kg/m^2^)	319 (33.9)	9 (23.1)	310 (34.4)	0.169
Underlying disease				
Hypertension, *n* (%)	712 (75.7)	30 (76.9)	682 (75.6)	1.000
Diabetes mellitus, *n* (%)	391 (41.6)	20 (51.3)	371 (41.1)	0.246
Dyslipidemia, *n* (%)	491 (52.2)	18 (46.2)	473 (52.4)	0.513
Hemodialysis, *n* (%)	44 (4.7)	1 (2.6)	43 (4.8)	1.000
Previous atrial fibrillation, *n* (%)	40 (4.3)	1 (2.6)	39 (4.3)	1.000
History of ischemic stroke or TIA, *n* (%)	88 (9.4)	7 (17.9)	81 (9)	0.083
History of peripheral artery disease, *n* (%)	31 (3.3)	2 (5.1)	29 (3.2)	0.371
History of previous PCI, *n* (%)	120 (12.8)	5 (12.8)	115 (12.7)	1.000
History of previous CABG, *n* (%)	13 (1.4)	0 (0)	13 (1.4)	1.000
History of previous MI, *n* (%)	95 (10.1)	5 (12.8)	90 (10)	0.583
Medication before admission				
Aspirin, *n* (%)	156/925 (16.9)	5/34 (14.7)	151/891 (16.9)	1.000
Thienopyridine, *n* (%)	80/924 (8.7)	3/34 (8.8)	77/890 (8.7)	1.000
Beta-blocker, *n* (%)	130/910 (14.3)	4/32 (12.5)	126/878 (14.4)	1.000
ACE-inhibitor, ARB, *n* (%)	291/910 (32)	9/32 (28.1)	282/878 (32.1)	0.704
Statin, *n* (%)	229/915 (25)	6/33 (18.2)	223/882 (25.3)	0.419
Hypoglycemic agents, *n* (%)	221/915 (24.2)	9/32 (28.1)	212/883 (24)	0.674
Insulin, *n* (%)	42/919 (4.6)	3/34 (8.8)	39/885 (4.4)	0.200
Warfarin, *n* (%)	12/926 (1.3)	1/34 (2.9)	11/892 (1.2)	0.363
DOAC, *n* (%)	20/926 (2.2)	1/34 (2.9)	19/892 (2.1)	0.531
Killip class				<0.001
1, *n* (%)	656 (69.7)	11 (28.2)	645 (71.5)	
2, *n* (%)	65 (6.9)	2 (5.1)	63 (7)	
3, *n* (%)	70 (7.4)	5 (12.8)	65 (7.2)	
4, *n* (%)	150 (15.9)	21 (53.8)	129 (14.3)	
Cardiogenic shock, *n* (%)	150 (15.9)	21 (53.8)	129 (14.3)	<0.001
OHCA, *n* (%)	62 (6.6)	13 (33.3)	49 (5.4)	<0.001
Laboratory data at arrival				
Estimated GFR (mL/min/1.73 m^2^)	65.1 (48.0–81.5)	47.6 (36.6–63.4)	65.9 (49.1–81.9)	<0.001
Hemoglobin (g/dL)	13.7 (12.2–15.0)	13.9 (12.2–15.2)	13.7 (12.2–15.0)	0.726
BNP (pg/mL)	103.2 (30.4–352.4) (*n* = 902)	177.9 (53.6–508.4) (*n* = 34)	101.9 (30.0–346.1) (*n* = 868)	0.095
New-onset AF during admission, *n* (%)	120 (12.8)	11 (28.2)	109 (12.1)	0.011
Left ventricular thrombus during admission, *n* (%)	27 (2.9)	2 (5.1)	25 (2.8)	0.309

Data are expressed as median and inter-quartile range, the mean ± standard deviation or number (percentage). Normally distributed continuous variables were compared by the Student’s *t*-test. Otherwise, continuous variables were compared by the Mann–Whitney U test. The Fischer exact test was used for categorical variables. Abbreviations: ACE = angiotensin converting enzyme, AF = atrial fibrillation, ARB = angiotensin II receptor blocker, AIS = acute ischemic stroke, BNP = brain natriuretic peptide, CABG = coronary artery bypass grafting, DOAC = direct oral anticoagulant, GFR = glomerular filtration rate, MI = myocardial infarction, OHCA = out-of-hospital cardiac arrest, PCI = percutaneous coronary intervention, TIA = transient ischemic attack.

**Table 2:** Comparison of lesion and procedural characteristics of primary PCI between the AIS/TIA group and the non-AIS/TIA group.

	All (*n* = 941)	AIS/TIA Group (*n* = 39)	Non-AIS/TIA Group (*n* = 902)	*p*-Value
Culprit lesion				0.152
LM-LAD, *n* (%)	488 (51.9)	20 (51.3)	468 (51.9)	
RCA, *n* (%)	361 (38.4)	13 (33.3)	348 (38.6)	
LCX, *n* (%)	89 (9.5)	5 (12.8)	84 (9.3)	
Graft, *n* (%)	3 (0.3)	1 (2.6)	2 (0.2)	
Triple vessels disease, *n* (%)	198 (21)	16 (41)	182 (20.2)	0.004
Anomalous origin of coronary artery, *n* (%)	24 (2.6)	1 (2.6)	23 (2.5)	1.000
Initial TIMI flow grade of culprit ≤2, *n* (%)	785 (83.4)	33 (84.6)	752 (83.4)	1.000
Final TIMI flow grade of culprit ≤2, *n* (%)	65 (6.9)	6 (15.4)	59 (6.5)	0.046
Lesion length (mm)	13.6 (9.4–19.8)	14.1 (7.1–21.8)	13.6 (9.5–19.6)	0.651
Reference diameter (mm)	2.4 (2.0–2.9)	2.4 (1.9–2.7)	2.4 (2.0–2.9)	0.540
Eccentricity, *n* (%)	267 (28.4)	5 (12.8)	262 (29)	0.029
Moderately–extremely angulated lesion, *n* (%)	92 (9.8)	7 (17.9)	85 (9.4)	0.094
Irregular contour, *n* (%)	586 (62.3)	23 (59)	563 (62.4)	0.736
Ostial lesion, *n* (%)	20 (2.1)	1 (2.6)	19 (2.1)	0.575
Bifurcation lesion, *n* (%)	182 (19.3)	4 (10.3)	178 (19.7)	0.211
Excessive tortuosity, *n* (%)	115 (12.2)	7 (17.9)	108 (12)	0.312
Moderate-severe calcification, *n* (%)	160 (17)	8 (20.5)	152 (16.9)	0.517
Thrombus (TIMI Thrombus grade ≥3), *n* (%)	414 (44)	15 (38.5)	399 (44.2)	0.514
ACC/AHA classification: type B2/C, *n* (%)	794 (84.4)	29 (74.4)	765 (84.8)	0.110
Approach site				0.001
Trans-radial approach, *n* (%)	637 (67.7)	14 (35.9)	623 (69.1)	
Trans-femoral approach, *n* (%)	292 (31)	25 (64.1)	267 (29.6)	
Trans-brachial approach, *n* (%)	12 (1.3)	0 (0)	12 (1.3)	
Number of used catheters	3 (3–3)	3 (3–4)	3 (3–3)	<0.001
Diagnostic catheters	2 (2–2)	2 (2–3)	2 (2–2)	0.001
Guiding catheters	1 (1–1)	1 (1–1)	1 (1–1)	0.001
Use of ≥4 catheters, *n* (%)	174 (18.5)	17 (43.6)	157 (17.4)	<0.001
Size of guiding catheter				0.001
6 Fr, *n* (%)	697 (74.1)	19 (48.7)	678 (75.2)	
7 Fr, *n* (%)	239 (25.4)	19 (48.7)	220 (24.4)	
8 Fr, *n* (%)	5 (0.5)	1 (0.6)	4 (0.4)	
Multivessel PCI at the time of primary PCI	19 (2)	5 (12.8)	14 (1.6)	0.001
Final PCI procedure				0.756
POBA only, *n* (%)	46 (4.9)	1 (2.6)	45 (5)	
Thrombus aspiration only, *n* (%)	11 (1.2)	1 (2.6)	10 (1.1)	
Thrombus aspiration and POBA, *n* (%)	11 (1.2)	0 (0)	11 (1.2)	
Bare metal stent, *n* (%)	17 (1.8)	0 (0)	17 (1.9)	
Drug-eluting stent, *n* (%)	831 (88.4)	37 (94.9)	794 (88.1)	
Drug-coated balloon, *n* (%)	24 (2.6)	0 (0)	24 (2.7)	
Bougie with micro-catheter, *n* (%)	1 (0.1)	0 (0)	1 (0.1)	
Thrombus aspiration procedure, *n* (%)	206 (21.9)	9 (23.1)	197 (21.8)	0.844
Use of guide extension catheters, *n* (%)	92 (9.8)	6 (15.4)	86 (9.5)	0.263
IABP, *n* (%)	96 (10.2)	7 (17.9)	89 (9.9)	0.106
V-A ECMO, *n* (%)	57 (6.1)	17 (43.6)	40 (4.4)	<0.001
Impella (Abiomed), *n* (%)	3 (0.3)	2 (5.1)	1 (0.1)	0.005
Door-to-balloon time (minutes)	72 (56–106)	97 (68–137)	71 (56–104)	0.006
Procedure time (minutes)	52 (40–73)	63 (49–96)	51 (40–72)	0.005
BARC type 3 or 5 bleeding, *n* (%)	69 (7.3)	12 (30.8)	57 (6.3)	<0.001
Discontinuation of anti-thrombotic therapy, *n* (%)	13 (1.4)	3 (7.7)	10 (1.1)	0.014

Data are expressed as median and inter-quartile range, the mean ± standard deviation or number (percentage). Normally distributed continuous variables were compared by the Student’s *t*-test. Otherwise, continuous variables were compared by the Mann–Whitney U test. The Fischer exact test was used for categorical variables. Abbreviations: ACC = American college of cardiology, AHA = American heart association, AIS = acute ischemic stroke, BARC= bleeding academic research consortium, IABP = intra-aortic balloon pumping, LCX = left circumflex artery, LM-LAD = left main-left anterior descending artery, PCI = percutaneous coronary intervention, POBA = percutaneous old balloon angioplasty, RCA = right coronary artery, TIA = transient ischemic attack, TIMI = Thrombolysis in myocardial infarction, V-A ECMO= veno-arterial extracorporeal membrane oxygenation.

**Table 3:** Comparison of outcomes and clinical course between the AIS/TIA group and the non-AIS/TIA group.

	All (*n* = 941)	AIS/TIA Group (*n* = 39)	Non-AIS/TIA Group (*n* = 902)	*p*-Value
In-hospital death, *n* (%)	75 (8)	18 (46.2)	57 (6.3)	<0.001
Among patients without cardiogenic shock (*n* = 791)	20/791 (2.5)	5/18 (27.8)	15/773 (1.9)	<0.001
Among patients with cardiogenic shock (*n* = 150)	55/150 (36.7)	13/21 (61.9)	42/129 (32.6)	0.014
Favorable neurological function (CPC 1 or 2) at discharge, *n* (%)	835 (88.7)	14 (35.9)	821 (91)	<0.001
Tracheostomy, *n* (%)	11 (1.2)	3 (7.7)	8 (0.9)	0.009
Mechanical ventilation (including NPPV), *n* (%)	223 (23.7)	30 (76.9)	193 (21.4)	<0.001
Ejection fraction at discharge (%)	52.2 (41.8–60.6) (*n* = 877)	42.5 (26.6–58.4) (*n* = 24)	52.5 (42.0–60.7) (*n* = 853)	0.021
Number of cardiac catheterizations during admission	1 (1–2)	1 (1–1)	1 (1–2)	0.298

Data are expressed as median and inter-quartile range, the mean ± standard deviation or number (percentage). Normally distributed continuous variables were compared by the Student’s *t*-test. Otherwise, continuous variables were compared by the Mann–Whitney U test. The Fischer exact test was used for categorical variables. Abbreviations: AIS = acute ischemic stroke, CPC = cerebral performance category, NPPV = non-invasive positive pressure ventilation, TIA = transient ischemic attack.

**Table 4:** Multivariate stepwise logistic regression models to investigate the factors associated with AIS/TIA.

Independent Variables	Dependent Variable: AIS/TIA					
	Model 1			Model 2		
	Odds Ratio	95% CI	*p*-Value	Odds Ratio	95% CI	*p*-Value
OHCA				5.126	2.169–12.113	<0.001
Cardiogenic shock	3.228	1.492–6.986	0.003			
New-onset AF	2.280	1.033–5.031	0.041	2.914	1.296–6.551	0.010
Lesion eccentricity	0.446	0.167–1.196	0.109	0.406	0.148–1.113	0.080
Final TIMI flow grade ≤2				2.490	0.911–6.811	0.075
Triple vessels disease				2.036	0.976–4.248	0.058
Trans-femoral approach	2.336	1.093–4.992	0.029	2.502	1.186–5.277	0.016
Use of ≥4 catheters	3.715	1.831–7.537	<0.001	3.460	1.660–7.212	0.001
BARC type 3 or 5 bleeding	2.932	1.256–6.846	0.013	2.958	1.254–6.975	0.013

In this stepwise model, 11 independent variables that showed significant difference (*p* < 0.05) between the AIS/TIA group and the non-AIS/TIA group in Table 1 and Table 2 were included. Model 1 included cardiogenic shock instead of OHCA and Model 2 included OHCA instead of cardiogenic shock. Likelihood ratio statistical criteria using the backward elimination method was performed. Abbreviations: AF = atrial fibrillation, AIS = acute ischemic stroke, BARC= bleeding academic research consortium, CI = confidence interval, OHCA = out-of-hospital cardiac arrest, TIA = transient ischemic attack, TIMI = Thrombolysis in myocardial infarction.

## Data Availability

All data are available from the corresponding author on reasonable request.

## Supplementary Materials

The following supporting information can be downloaded at: https://www.mdpi.com/article/10.3390/jcm12030840/s1, Table S1: Characteristics of 39 AIS/TIA patients, Table S2: Comparisons of the clinical characteristics and outcomes between the early-onset AIS/TIA group and the late-onset AIS/TIA group.

## References

[1] Hachet O., Guenancia C., Stamboul K., Daubail B., Richard C., Bejot Y., Yameogo V., Gudjoncik A., Cottin Y., Giroud M. (2014). Frequency and predictors of stroke after acute myocardial infarction: Specific aspects of in-hospital and postdischarge events. Stroke.

[2] Aggarwal G., Patlolla S.H., Aggarwal S., Cheungpasitporn W., Doshi R., Sundaragiri P.R., Rabinstein A.A., Jaffe A.S., Barsness G.W., Cohen M. (2021). Temporal Trends, Predictors, and Outcomes of Acute Ischemic Stroke in Acute Myocardial Infarction in the United States. J. Am. Heart Assoc..

[3] Alkhouli M., Alqahtani F., Tarabishy A., Sandhu G., Rihal C.S. (2019). Incidence, Predictors, and Outcomes of Acute Ischemic Stroke Following Percutaneous Coronary Intervention. JACC Cardiovasc. Interv..

[4] Kajermo U., Ulvenstam A., Modica A., Jernberg T., Mooe T. (2014). Incidence, trends, and predictors of ischemic stroke 30 days after an acute myocardial infarction. Stroke.

[5] Al Suwaidi J., Al Habib K., Asaad N., Singh R., Hersi A., Al Falaeh H., Al Saif S., Al-Motarreb A., Almahmeed W., Sulaiman K. (2012). Immediate and one-year outcome of patients presenting with acute coronary syndrome complicated by stroke: Findings from the 2nd Gulf Registry of Acute Coronary Events (Gulf RACE-2). BMC Cardiovasc. Disord..

[6] Patil S., Gonuguntla K., Rojulpote C., Kumar M., Nadadur S., Nardino R.J., Pickett C. (2021). Prevalence and Determinants of Atrial Fibrillation-Associated In-Hospital Ischemic Stroke in Patients with Acute Myocardial Infarction Undergoing Percutaneous Coronary Intervention. Am. J. Cardiol..

[7] Sotomi Y., Ueda Y., Hikoso S., Nakatani D., Suna S., Dohi T., Mizuno H., Okada K., Kida H., Oeun B. (2021). Manual Thrombus Aspiration and its Procedural Stroke Risk in Myocardial Infarction. J. Am. Heart Assoc..

[8] Shoji S., Kohsaka S., Kumamaru H., Sawano M., Shiraishi Y., Ueda I., Noma S., Suzuki M., Numasawa Y., Hayashida K. (2018). Stroke after Percutaneous Coronary Intervention in the Era of Transradial Intervention. Circ. Cardiovasc. Interv..

[9] Fuchs S., Stabile E., Kinnaird T.D., Mintz G.S., Gruberg L., Canos D.A., Pinnow E.E., Kornowski R., Suddath W.O., Satler L.F. (2002). Stroke complicating percutaneous coronary interventions: Incidence, predictors, and prognostic implications. Circulation.

[10] Ibanez B., James S., Agewall S., Antunes M.J., Bucciarelli-Ducci C., Bueno H., Caforio A.L.P., Crea F., Goudevenos J.A., Halvorsen S. (2018). 2017 ESC Guidelines for the management of acute myocardial infarction in patients presenting with ST-segment elevation: The Task Force for the management of acute myocardial infarction in patients presenting with ST-segment elevation of the European Society of Cardiology (ESC). Eur. Heart J..

[11] O’Gara P.T., Kushner F.G., Ascheim D.D., Casey D.E., Jr Chung M.K., de Lemos J.A., Ettinger S.M., Fang J.C., Fesmire F.M., Franklin B.A. (2013). 2013 ACCF/AHA guideline for the management of ST-elevation myocardial infarction: A report of the American College of Cardiology Foundation/American Heart Association Task Force on Practice Guidelines. Circulation.

[12] Taguchi Y., Kubo S., Ikuta A., Osakada K., Takamatsu M., Takahashi K., Ohya M., Shimada T., Miura K., Murai R. (2022). Percutaneous coronary intervention for left main coronary artery malperfusion in acute type A aortic dissection. Cardiovasc. Interv. Ther..

[13] Mendelson S.J., Prabhakaran S. (2021). Diagnosis and Management of Transient Ischemic Attack and Acute Ischemic Stroke: A Review. JAMA.

[14] Ishibashi S., Sakakura K., Asada S., Taniguchi Y., Jinnouchi H., Tsukui T., Yamamoto K., Seguchi M., Wada H., Fujita H. (2021). Factors associated with difficulty in crossing the culprit lesion of acute myocardial infarction. Sci. Rep..

[15] Thygesen K., Alpert J.S., Jaffe A.S., Chaitman B.R., Bax J.J., Morrow D.A., White H.D. (2018). Fourth Universal Definition of Myocardial Infarction (2018). J. Am. Coll. Cardiol..

[16] Murakami T., Sakakura K., Taniguchi Y., Yamamoto K., Tsukui T., Seguchi M., Jinnouchi H., Wada H., Fujita H. (2022). Comparison of the cost in percutaneous coronary intervention between ST-segment elevation myocardial infarction vs. non-ST-segment elevation myocardial infarction. Cardiovasc. Interv. Ther..

[17] Kobayashi S., Sakakura K., Jinnouchi H., Taniguchi Y., Tsukui T., Watanabe Y., Yamamoto K., Seguchi M., Wada H., Fujita H. (2022). Comparison of door-to-balloon time and in-hospital outcomes in patients with ST-elevation myocardial infarction between before versus after COVID-19 pandemic. Cardiovasc. Interv. Ther..

[18] Taniguchi Y., Sakakura K., Adachi Y., Akashi N., Watanabe Y., Noguchi M., Yamamoto K., Ugata Y., Wada H., Momomura S.I. (2018). In-hospital outcomes of acute myocardial infarction with cardiogenic shock caused by right coronary artery occlusion vs. left coronary artery occlusion. Cardiovasc. Interv. Ther..

[19] Mehran R., Rao S.V., Bhatt D.L., Gibson C.M., Caixeta A., Eikelboom J., Kaul S., Wiviott S.D., Menon V., Nikolsky E. (2011). Standardized bleeding definitions for cardiovascular clinical trials: A consensus report from the Bleeding Academic Research Consortium. Circulation.

[20] Yasuda S., Honda S., Takegami M., Nishihira K., Kojima S., Asaumi Y., Suzuki M., Kosuge M., Takahashi J., Sakata Y. (2019). Contemporary Antiplatelet Therapy and Clinical Outcomes of Japanese Patients with Acute Myocardial Infarction—Results from the Prospective Japan Acute Myocardial Infarction Registry (JAMIR). Circ. J..

[21] Murakami T., Sakakura K., Jinnouchi H., Taniguchi Y., Tsukui T., Watanabe Y., Yamamoto K., Seguchi M., Wada H., Fujita H. (2022). Comparison of medical resource use and total admission cost in patients with acute myocardial infarction between on-hours visit versus off-hours visit. Cardiovasc. Interv. Ther..

[22] Watanabe Y., Sakakura K., Taniguchi Y., Yamamoto K., Seguchi M., Tsukui T., Jinnouchi H., Wada H., Fujita H. (2022). Long-term outcomes of the modest stent expansion strategy for the culprit lesion of acute myocardial infarction. Cardiovasc. Interv. Ther..

[23] Naito R., Sakakura K., Wada H., Funayama H., Sugawara Y., Kubo N., Ako J., Momomura S. (2012). Comparison of long-term clinical outcomes between sirolimus-eluting stents and paclitaxel-eluting stents following rotational atherectomy. Int. Heart J..

[24] Yildiz A., Okcun B., Peker T., Arslan C., Olcay A., Bulent Vatan M. (2010). Prevalence of coronary artery anomalies in 12,457 adult patients who underwent coronary angiography. Clin. Cardiol..

[25] Guptill J.T., Mehta R.H., Armstrong P.W., Horton J., Laskowitz D., James S., Granger C.B., Lopes R.D. (2013). Stroke after primary percutaneous coronary intervention in patients with ST-segment elevation myocardial infarction: Timing, characteristics, and clinical outcomes. Circ. Cardiovasc. Interv..

[26] Takeji Y., Shiomi H., Morimoto T., Yoshikawa Y., Taniguchi R., Mutsumura-Nakano Y., Yamamoto K., Yamaji K., Tazaki J., Kato E.T. (2021). Changes in demographics, clinical practices and long-term outcomes of patients with ST segment-elevation myocardial infarction who underwent coronary revascularisation in the past two decades: Cohort Study. BMJ Open.

[27] Murai M., Hazui H., Sugie A., Hoshiga M., Negoro N., Muraoka H., Miyamoto H., Kobata H., Fukumoto H., Ishihara T. (2008). Asymptomatic acute ischemic stroke after primary percutaneous coronary intervention in patients with acute coronary syndrome might be caused mainly by manipulating catheters or devices in the ascending aorta, regardless of the approach to the coronary artery. Circ. J..

[28] Korn-Lubetzki I., Farkash R., Pachino R.M., Almagor Y., Tzivoni D., Meerkin D. (2013). Incidence and risk factors of cerebrovascular events following cardiac catheterization. J. Am. Heart Assoc..

[29] Varmdal T., Janszky I., Bakken I.J., Ellekjaer H., Fjaertoft H., Haberg S.E., Bonaa K.H. (2017). Percutaneous Coronary Intervention as a Trigger for Stroke. Am. J. Cardiol..

[30] Chandiramani R., Chen H., Aoi S., Giustino G., Claessen B., Sartori S., Aquino M., Sorrentino S., Cao D., Goel R. (2020). Incidence, predictors and impact of stroke on mortality among patients with acute coronary syndromes following percutaneous coronary intervention-Results from the PROMETHEUS registry. Catheter. Cardiovasc. Interv..

[31] Hoffman S.J., Routledge H.C., Lennon R.J., Mustafa M.Z., Rihal C.S., Gersh B.J., Holmes D.R., Gulati R. (2012). Procedural factors associated with percutaneous coronary intervention-related ischemic stroke. JACC Cardiovasc. Interv..

[32] Sharma U., Tak T. (2011). Aortic atheromas: Current knowledge and controversies: A brief review of the literature. Echocardiography.

[33] Harloff A., Simon J., Brendecke S., Assefa D., Helbing T., Frydrychowicz A., Weber J., Olschewski M., Strecker C., Hennig J. (2010). Complex plaques in the proximal descending aorta: An underestimated embolic source of stroke. Stroke.

[34] Keeley E., Grines C.L. (1998). Scraping of aortic debris by coronary guiding catheters. J. Am. Coll. Cardiol..

[35] Eggebrecht H., Oldenburg O., Dirsch O., Haude M., Baumgart D., Welge D., Herrmann J., Arnold G., Schmid K.W., Erbel R. (2000). Potential embolization by atherosclerotic debris dislodged from aortic wall during cardiac catheterization:: Histological and clinical findings in 7,621 patients. Catheter. Cardiovasc. Interv..

[36] Joshi N.V., Toor I., Shah A.S., Carruthers K., Vesey A.T., Alam S.R., Sills A., Hoo T.Y., Melville A.J., Langlands S.P. (2015). Systemic Atherosclerotic Inflammation Following Acute Myocardial Infarction: Myocardial Infarction Begets Myocardial Infarction. J. Am. Heart Assoc..

[37] Le May M., Wells G., So D., Chong A.Y., Dick A., Froeschl M., Glover C., Hibbert B., Marquis J.F., Blondeau M. (2020). Safety and Efficacy of Femoral Access vs. Radial Access in ST-Segment Elevation Myocardial Infarction: The SAFARI-STEMI Randomized Clinical Trial. JAMA Cardiol..

[38] Blackshear J.L., Pearce L.A., Hart R.G., Zabalgoitia M., Labovitz A., Asinger R.W., Halperin J.L. (1999). Aortic plaque in atrial fibrillation: Prevalence, predictors, and thromboembolic implications. Stroke.

[39] Craiem D., Chironi G., Casciaro M.E., Graf S., Simon A. (2014). Calcifications of the thoracic aorta on extended non-contrast-enhanced cardiac CT. PLoS ONE.

[40] Kojima K., Kimura S., Hayasaka K., Mizusawa M., Misawa T., Yamakami Y., Sagawa Y., Ohtani H., Hishikari K., Sugiyama T. (2019). Aortic Plaque Distribution, and Association between Aortic Plaque and Atherosclerotic Risk Factors: An Aortic Angioscopy Study. J. Atheroscler. Thromb..

[41] Kundu A., O’Day K., Shaikh A.Y., Lessard D.M., Saczynski J.S., Yarzebski J., Darling C.E., Thabet R., Akhter M.W., Floyd K.C. (2016). Relation of Atrial Fibrillation in Acute Myocardial Infarction to In-Hospital Complications and Early Hospital Readmission. Am. J. Cardiol..

[42] Natsuaki M., Morimoto T., Yamaji K., Watanabe H., Yoshikawa Y., Shiomi H., Nakagawa Y., Furukawa Y., Kadota K., Ando K. (2018). Prediction of Thrombotic and Bleeding Events after Percutaneous Coronary Intervention: CREDO-Kyoto Thrombotic and Bleeding Risk Scores. J. Am. Heart Assoc..

[43] Powers W.J., Rabinstein A.A., Ackerson T., Adeoye O.M., Bambakidis N.C., Becker K., Biller J., Brown M., Demaerschalk B.M., Hoh B. (2018). 2018 Guidelines for the Early Management of Patients with Acute Ischemic Stroke: A Guideline for Healthcare Professionals from the American Heart Association/American Stroke Association. Stroke.

[44] Lee K.H., Torii S., Oguri M., Miyaji T., Kiyooka T., Ono Y., Asada K., Adachi T., Takahashi A., Ikari Y. (2022). Reduction of door-to-balloon time in patients with ST-elevation myocardial infarction by single-catheter primary percutaneous coronary intervention method. Catheter. Cardiovasc. Interv..

[45] Akashi N., Sakakura K., Yamamoto K., Taniguchi Y., Wada H., Momomura S.I., Fujita H. (2017). Minimization of door-to-balloon time for ST-elevation acute myocardial infarction: A case report. Clin. Case Rep..

[46] Ahsan M.J., Ahmad S., Latif A., Lateef N., Ahsan M.Z., Abusnina W., Nathan S., Altin S.E., Kolte D.S., Messenger J.C. (2022). Transradial versus transfemoral approach for percutaneous coronary intervention in patients with ST-elevation myocardial infarction complicated by cardiogenic shock: A systematic review and meta-analysis. Eur. Heart J. Qual. Care Clin. Outcomes.

[47] Kashiwagi M., Tanimoto T., Kitabata H., Arita Y., Yamamoto Y., Mori K., Terada K., Nishiguchi T., Taruya A., Kubo T. (2019). Usefulness of rescue ultrasound guidance for transradial cardiac catheterization. Cardiovasc. Revasc. Med..

[48] Wernly B., Seelmaier C., Leistner D., Stahli B.E., Pretsch I., Lichtenauer M., Jung C., Hoppe U.C., Landmesser U., Thiele H. (2019). Mechanical circulatory support with Impella versus intra-aortic balloon pump or medical treatment in cardiogenic shock-a critical appraisal of current data. Clin. Res. Cardiol..

