# Supplementary material for: Acute Ischemic Stroke and Transient Ischemic Attack in ST-Segment Elevation Myocardial Infarction Patients Who Underwent Primary Percutaneous Coronary Intervention
Source: J Clin Med. 2023 Jan 20;12(3):840. doi: 10.3390/jcm12030840 (PMC9917385; doi:10.3390/jcm12030840)
Supplement: Supplementary file 1 [file jcm-12-00840-s001.zip › jcm-2122875-SI.pdf]

Journal of Clinical Medicine

Supplementary Materials

Table S1. Characteristics of 39 AIS/TIA patients.

Characteristics	Number of Patients (%)
Stroke type	
Acute ischemic stroke	36 (92.3)
Transient ischemic attack	3 (7.7)
Clinical symptom	
Consciousness disorder	24 (61.5)
Hemiparesis	17 (43.6)
Aphasia	3 (7.7)
Hypesthesia	1 (2.6)
Hemispatial neglect	1 (2.6)
Visual field defect	1 (2.6)
Headache	1 (2.6)
Vascular territory determined by cerebral imaging studies	
Anterior cerebral artery	7 (17.9)
Middle cerebral artery	27 (69.2)
Posterior cerebral artery	10 (25.6)
Vertebrobasilar artery	12 (30.8)
Multiple cerebral infarction	19 (48.7)
Cerebral imaging studies could not detect acute ischemic cerebral infarction	3 (7.7)

Abbreviations: AIS = acute ischemic stroke, TIA = transient ischemic attack.

Table S2. Comparisons of the clinical characteristics and outcomes between the early-onset AIS/TIA group and the late-onset AIS/TIA group.

	All (*n* = 39)	Early-Onset AIS/TIA (*n* = 16)	Late-Onset AIS/TIA (*n* = 23)	*p*-Value
Age, year	74 (69–77)	75 (72–78)	71 (63–77)	0.157
Male, *n* (%)	33 (84.6)	13 (81.2)	20 (87)	0.674
Body mass index >25 (kg/m²)	9 (21.7)	4 (25)	5 (21.7)	1.000
Underlying disease				
Hypertension, *n* (%)	30 (76.9)	12 (75)	18 (78.3)	1.000
Diabetes mellitus, *n* (%)	20 (51.3)	8 (50)	12 (52.2)	1.000
Dyslipidemia, *n* (%)	18 (46.2)	7 (43.8)	11 (47.8)	1.000
Hemodialysis, *n* (%)	1 (2.6)	0 (0)	1 (4.3)	1.000
Previous atrial fibrillation, *n* (%)	1 (2.6)	1 (6.2)	0 (0)	0.410
History of ischemic stroke or TIA, *n* (%)	7 (17.9)	4 (25)	3 (13)	0.415
History of peripheral artery disease, *n* (%)	2 (5.1)	0 (0)	2 (8.7)	0.503
History of previous PCI, *n* (%)	5 (12.8)	4 (25)	1 (4.3)	0.139
History of previous CABG, *n* (%)	0 (0)	0 (0)	0 (0)	
History of previous MI, *n* (%)	5 (12.8)	4 (25)	1 (4.3)	0.139
Medication before admission				
Aspirin, *n* (%)	5/34 (14.7)	1/12 (8.3)	4/22 (18.2)	0.635
Thienopyridine, *n* (%)	3/34 (8.8)	2/12 (16.7)	1/22 (4.5)	0.279
Beta-blocker, *n* (%)	4/32 (12.5)	1/12 (8.3)	3/20 (15)	1.000
ACE-inhibitor, ARB, *n* (%)	9/32 (28.1)	3/12 (25)	6/20 (30)	1.000
Statin, *n* (%)	6/33 (18.2)	4/12 (33.3)	2/21 (9.5)	0.159
Hypoglycemic agents, *n* (%)	9/32 (28.1)	3/12 (25)	6/20 (30)	1.000
Insulin, *n* (%)	3/34 (8.8)	0/12 (0)	3/22 (13.6)	0.537
Warfarin, *n* (%)	1/34 (2.9)	1/12 (8.3)	0/22 (0)	0.353
DOAC, *n* (%)	1/34 (2.9)	0/12 (0)	1/22 (4.5)	1.000

Killip class ≥2, *n* (%)	18 (46.2)	11 (68.8)	17 (73.9)	0.734
Cardiogenic shock, *n* (%)	21 (53.8)	9 (56.2)	12 (52.2)	1.000
Laboratory data at arrival				
Estimated GFR (ml/min/1.73 m^2)	47.6 (37.1–60.7)	46.9 (38.4–57.7)	48.0 (27.7–70.3)	0.886
Hemoglobin (g/dl)	13.9 (12.4–15.1)	13.4 (12.1–14.5)	14.4 (12.5–15.5)	0.361
BNP (pg/mL)	177.9 (53.6–508.4) (*n* = 34)	112.1 (25–425.3) (*n* = 13)	253.5 (53.8–582.6) (*n* = 21)	0.425
New-onset AF during admission, *n* (%)	11 (28.2)	3 (18.8)	8 (34.8)	0.471
Left ventricular thrombus during admission, *n* (%)	2 (5.1)	0 (0)	2 (8.7)	0.503
Culprit lesion				0.761
LM-LAD, *n* (%)	20 (51.3)	7 (43.8)	13 (56.5)	
RCA, *n* (%)	13 (33.3)	6 (37.5)	7 (30.4)	
LCX, *n* (%)	5 (12.8)	2 (12.5)	3 (13)	
Graft, *n* (%)	1 (2.6)	1 (6.2)	0 (0)	
Triple vessels disease, *n* (%)	16 (41)	6 (37.5)	10 (43.5)	0.752
Anomalous origin of coronary artery, *n* (%)	1 (2.6)	0 (0)	1 (4.3)	1.000
Initial TIMI flow grade of culprit ≤2, *n* (%)	33 (84.6)	13 (81.2)	20 (87)	0.674
Final TIMI flow grade of culprit ≤2, *n* (%)	6 (15.4)	3 (18.8)	3 (13)	0.674
Lesion length (mm)	14.1 (7.4–21.4)	13.4 (6.6–22.4)	15.0 (7.8–20.9)	0.607
Reference diameter (mm)	2.4 (2.0–2.6)	2.5 (2.0–2.8)	2.3 (1.9–2.6)	0.466
Eccentricity, *n* (%)	5 (12.8)	2 (12.5)	3 (13)	1.000
Moderately-extremely angulated lesion, *n* (%)	7 (17.9)	4 (25)	3 (13)	0.415
Irregular contour, *n* (%)	23 (59)	8 (50)	15 (65.2)	0.509
Ostial lesion, *n* (%)	1 (2.6)	1 (6.2)	0 (0)	0.410
Bifurcation lesion, *n* (%)	4 (10.3)	2 (12.5)	2 (8.7)	1.000
Excessive tortuosity, *n* (%)	7 (17.9)	4 (25)	3 (13)	0.415
Moderate-severe calcification, *n* (%)	8 (20.5)	4 (25)	4 (17.4)	0.694
Thrombus (TIMI Thrombus grade ≥3), *n* (%)	15 (38.5)	7 (43.8)	8 (34.8)	0.740
ACC/AHA classification: type B2/C, *n* (%)	29 (74.4)	12 (75)	17 (73.9)	1.000
Approach site				1.000
Trans-radial approach, *n* (%)	14 (35.9)	6 (37.5)	8 (34.8)	
Trans-femoral approach, *n* (%)	25 (64.1)	10 (62.5)	15 (65.2)	
Trans-brachial approach, *n* (%)	0 (0)	0 (0)	0 (0)	
Number of used catheters	3 (3–4)	4 (3–4)	3 (3–4)	0.048
Diagnostic catheters	2 (2–3)	2 (2–3)	2 (2–2)	0.084
Guiding catheters	1 (1–1)	1 (1–2)	1 (1–1)	0.319
Use of ≥4 catheters, *n* (%)	17 (43.6)	10 (62.5)	7 (30.4)	0.059
Size of guiding catheter				0.849
6 Fr, *n* (%)	19 (48.7)	7 (43.8)	12 (52.2)	
7 Fr, *n* (%)	19 (48.7)	9 (56.2)	10 (43.5)	
8 Fr, *n* (%)	1 (2.6)	0 (0)	1 (4.3)	
Multivessel PCI at the time of primary PCI	5 (12.8)	2 (12.5)	3 (13)	1.000
Final PCI procedure				0.659
POBA only, *n* (%)	1 (2.6)	0 (0)	1 (4.3)	
Thrombus aspiration only, *n* (%)	1 (2.6)	1 (6.2)	0 (0)	
Thrombus aspiration and POBA, *n* (%)	0 (0)	0 (0)	0 (0)	
Bare metal stent, *n* (%)	0 (0)	0 (0)	0 (0)	
Drug-eluting stent, *n* (%)	37 (94.9)	15 (93.8)	22 (95.7)	
Drug coated balloon, *n* (%)	0 (0)	0 (0)	0 (0)	
Bougie with micro-catheter, *n* (%)	0 (0)	0 (0)	0 (0)	
Thrombus aspiration procedure, *n* (%)	9 (23.1)	3 (18.8)	6 (26.1)	0.711
Use of guide extension catheters, *n* (%)	6 (15.4)	3 (18.8)	3 (13)	0.674
IABP, *n* (%)	7 (17.9)	4 (25)	3 (13)	0.415
V-A ECMO, *n* (%)	17 (17.9)	6 (37.5)	11 (47.8)	0.743
Impella (Abiomed), *n* (%)	2 (5.1)	1 (6.2)	1 (4.3)	1.000
Door to balloon time (minutes)	97 (70–134)	106 (81–160)	91 (56–131)	0.170
Procedure time (minutes)	63 (50–94)	64 (50–101)	63 (39–85)	0.668
BARC type 3 or 5 bleeding, *n* (%)	12 (30.8)	4 (25)	8 (34.8)	0.726

Discontinuation of anti-thrombotic therapy, *n* (%)	3 (7.7)	1 (6.3)	2 (8.7)	1.000
Outcomes				
In-hospital death, *n* (%)	18 (46.2)	9 (56.2)	9 (39.1)	0.342
Favorable neurological function (CPC 1 or 2) at discharge, *n* (%)	14 (35.9)	5 (31.2)	9 (39.1)	0.740
Tracheostomy, *n* (%)	3 (7.7)	1 (6.2)	2 (8.7)	1.000
Mechanical ventilation (including NPPV), *n* (%)	30 (76.9)	11 (68.8)	19 (82.6)	0.444
Ejection fraction at discharge (%)	42.5 (26.6–58.4) (*n* = 24)	38.7 (27.0–65.0) (*n* = 9)	56.1 (46.3–62.1) (*n* = 15)	0.907
Number of cardiac catheterizations during admission	1 (1–1)	1 (1–1)	1 (1–1)	0.114

Data are expressed as median and inter-quartile range, the mean ±standard deviation or number (percentage). Normally-distributed continuous variables were compared by the Student's *t*-test. Otherwise, continuous variables were compared by the Mann-Whitney U test. Fischer exact test was used for categorical variables. Abbreviations: ACC = American college of cardiology, ACE = angiotensin converting enzyme, AF = atrial fibrillation, AHA = American heart association, ARB = angiotensin II receptor blocker, AIS = acute ischemic stroke, BARC= bleeding academic research consortium, BNP = brain natriuretic peptide, CABG = coronary artery bypass grafting, CPC = cerebral performance category, DOAC = direct oral anticoagulant, GFR = glomerular filtration rate, IABP = intra-aortic balloon pumping, LCX = left circumflex artery, LM-LAD = left main-left anterior descending artery, MI = myocardial infarction, NPPV = non-invasive positive pressure ventilation, PCI = percutaneous coronary intervention, POBA = percutaneous old balloon angioplasty, RCA = right coronary artery, TIA = transient ischemic attack, TIMI = Thrombolysis in myocardial infarction, V-A ECMO = veno-arterial extracorporeal membrane oxygenation.
